# Multidrug-resistant gram-negative bacterial infections in a teaching hospital in Ghana

**DOI:** 10.1186/s13756-018-0324-2

**Published:** 2018-03-09

**Authors:** Nicholas Agyepong, Usha Govinden, Alex Owusu-Ofori, Sabiha Yusuf Essack

**Affiliations:** 1 Antimicrobial Research Unit, Discipline of Pharmaceutical Sciences, University of Kwa-Zulu Natal, Durban, South Africa; 2 School of Medical Sciences, Kwame Nkrumah University of Science and Technology, Kumasi, Ghana

**Keywords:** Antibiotic resistance, Infections, Multidrug resistance, Pathogens

## Abstract

**Background:**

Multidrug-resistant Gram-negative bacteria have emerged as major clinical and therapeutic dilemma in hospitals in Ghana.

To describe the prevalence and profile of infections attributable to multidrug-resistant Gram-negative bacteria among patients at the Komfo Anokye Teaching Hospital in the Ashanti region of Ghana.

**Methods:**

Bacterial cultures were randomly selected from the microbiology laboratory from February to August, 2015. Bacterial identification and minimum inhibitory concentrations were conducted using standard microbiological techniques and the Vitek-2 automated system. Patient information was retrieved from the hospital data.

**Results:**

Of the 200 isolates, consisting of *K. pneumoniae*, *A. baumannii*, *P. aeruginosa*, *Enterobacter spp.*, *E. coli*, *Yersinia spp.*, *Proteus mirabilis*, *Pasteurella spp., Chromobacterium violaceum, Salmomella enterica*, *Vibrio spp.*, *Citrobacter koseri*, *Pantoea spp.*, *Serratia spp.*, *Providencia rettgeri Burkholderia cepacia*, *Aeromonas spp.*, *Cadecea lapagei* and *Sphingomonas paucimobilis*, 101 (50.5%) and 99 (49.5%) recovered from male and female patients respectively The largest proportion of patients were from age-group ≥60 years (24.5%) followed by < 10 years (24.0%) and least 10–19 years (9.5%) with a mean patient age of 35.95 ± 27.11 (0.2–91) years. The decreasing order of specimen source was urine 97 (48.5%), wound swabs 47 (23.5%), sputum 22 (11.0%) bronchial lavage, nasal and pleural swabs 1 (0.50%). Urinary tract infection was diagnosed in 34.5% of patients, sepsis in 14.5%, wound infections (surgical and chronic wounds) in 11.0%, pulmonary tuberculosis in 9.0% and appendicitis, bacteremia and cystitis in 0.50%. The isolates showed high resistance to ampicillin (94.4%), trimethoprim/sulfamethoxazole (84.5%), cefuroxime (79.0%) and cefotaxime (71.3%) but low resistance to ertapenem (1.5%), meropenem (3%) and amikacin (11%). The average multi-drug resistance was 89.5%, and ranged from 53.8% in *Enterobacter* spp. to 100.0% in *Acinetobacter* spp. and *P. aeruginosa*.

**Conclusion:**

Bacterial infections caused by multi-drug resistant (isolates resistant to at least one agent in three or more antibiotic classes) Gram-negative pathogens among patients at Komfo Anokye Teaching Hospital in Kumasi, Ghana are rife and interventions are necessary for their containment.

## Background

The emergence of multidrug-resistant Gram-negative bacteria is a major concern in hospital settings in many parts of the world. Infections caused by these pathogens have become significantly challenging over the past two decades, particularly in the developing countries, and are associated with high morbidity and mortality rates as well as protracted hospital stay [1]. Enterobacteriaceae including *Klebsiella pneumoniae*, *Escherichia coli* as well as *Enterobacter spp.* and non-lactose fermenting bacteria such as *Pseudomonas aeruginosa* and *Acinetobacter spp.* have been identified as major cause of multi-drug resistant bacterial infections [2–4].

Studies conducted in many developing countries including Africa, have indicated high antibiotic resistance among Gram-negative bacteria to commonly used antibiotics, leading to a loss of efficacy for treatment of common infections [5–7]. These resistant bacterial pathogens are a major cause of both community and hospital-acquired infections. Respiratory tract, urinary tract, bloodstream (septic), post-surgical (wound) infections and pneumonia are among most commonly reported infections attributable to these pathogens in many hospitals [8].

Although, the impact of antibiotic resistance caused by multidrug resistant Gram-negative bacteria has been recognised in hospitals in Ghana, measures such as surveillance studies that provide reliable data to mitigate the problem are not in place. Therefore studies to establish the prevalence and extent of resistance are necessary to bridge the information gap and provide the basis to guide empiric therapy. This study aimed to assess multidrug-resistance among Gram-negative bacteria in Komfo Anokye Teaching Hospital in Ghana to guide treatment protocols. The data further provides a baseline for future comparative studies.

## Materials

### Setting of study

The study was conducted between February and August 2015, in Komfo Anokye Teaching Hospital (KATH) in Kumasi, in the Ashanti region of Ghana. The facility is a 1000-bed tertiary care government hospital. The average daily primary care and specialist outpatient attendance was 169 and 954 patients respectively, during the period of study. The population of the region is concentrated in a few districts, with the Kumasi metropolis accounting for nearly one-third of the region’s population of 4,780,380 [9]. KATH is the only regional and referral hospital that takes care of about 80% of both emergencies and regular medical cases in the region and serves as referral hospital for part of Brong Ahafo, Western, Eastern and the Northern regions of Ghana.

### Bacterial collection and identification

Two hundred (200) clinical, non-duplicate Gram-negative bacteria were randomly selected from urine, pus, wound swab, pleural fluid endotracheal tubes, gastric lavage and blood specimens processed by the diagnostic microbiological laboratory in the hospital from both in-patients and out-patients. Information on diagnosis, sex, age and ward type were obtained from patients records. The isolates were maintained on nutrient agar slants, frozen in lyophilizing medium at − 70 °C and subsequently transported to National Health Laboratory Services in South Africa to confirm identification and ascertain antibiotic susceptibility profiles using Vitek-2 (Biomerieux, France) Automated Systems with *P. aeruginosa* ATCC27853 and *E.coli* ATCC35218 as control strains. Multidrug-resistance in this study was defined as isolates that were resistant to at least one agent in three or more antibiotic classes.

## Results

Of the 200 resistant Gram-negative bacterial isolates obtained, *E. coli* was most frequent pathogen 49 (24.5%), followed by *P. aeruginosa* 39 (19.5%), *K. pneumoniae* 38 (19.0%), *Enterobacter spp*. 12 (6.0%) *Serratia spp.* 8 (4.0%), *Sphingimonas spp.* 10 (5.0%) and *Acinetobacter spp.* 8 (4.0%). The remaining 36 (18%) (Table 1) consisted of *Yersinia spp.* (5) *Proteus mirabilis* (1), *Salmomella enterica* (1), *Vibrio spp.* (5), *Citrobacter koseri* (3), *Pantoea* spp. (5), *Providencia rettgeri* (4), *Pasteurella spp.* (2) *Chromobacterium violaceum* (2), *Burkholderia cepacia* (2), *Aeromonas spp.* (5), *Cadecea lapagei* (1). The distribution of the specimen types showed highest proportion of isolates, were from urine specimens 94 (47.0%), followed by wound swabs 45 (22.5%) and sputum 24 (12.0%) with just 1 (0.5%) from each of bronchial lavage, nasal and pleural swabs respectively. A higher proportion of isolates were recovered from in-patients 161 (80.5%) compared to the out-patients 39 (19.5%). The number of isolates from the medical intensive care unit (ICU) 78 (39.0%) was highest, followed by Child Health 39 (19.5%) and the Obstetrics and Gynecology wards 17 (8.5%) with the smallest number of 10 (5.0%) isolated in the Accident and Emergency Unit. *E. coli* 49 (24.5%), *P. aeruginosa* 39 (19.5%) and *K. pneumoniae* 38 (19.0%) (Table 1) were the most predominant pathogens implicated in 63.0% of all infections. The distribution of bacterial pathogens among clinical diagnosis showed urinary tract infection (UTI) 69 (34.5%) as the most prevalent, followed by sepsis 29 (14.5%), tuberculosis 18 (9.0%) and wound infections 12 (6.0%) with the least prevalence in appendicitis, bacteremia, cystitis and prostitis 1 (0.5%) (Table 1). *E. coli* 24 (34.8) was identified as most common cause of UTI, followed by *K. pneumoniae* 17 (24.6%) and *P. aeruginosa* 9 (13.04%) implicated in 72.4% of all UTI pathogens.

**Table 1:** Distribution of isolates among clinical specimen, wards and diagnosis

	Isolates (*N*)								
	*Acinetobacter spp*	*E. coli*	*Enterobacter spp*	*K. pneumonia*	*P. aeruginosa*	*Serretia spp*	*Sphingimonas spp*	Other*	Total (%)
Specimen									
Asctic fluid	–	–	–	–	–	–	–	1	1 (0.5)
Aspirate	–	2	1	1	–	–	–	–	4 (2.0)
Blood	–	1	2	2	–	–	–	6	11 (5.5)
Bronchial Lavage	–	–	1	–	–	–	–	–	1 (0.5)
Ear Swab	–	1	–	1	1	1	–	2	6 (3.0)
Gastric Lavage	–	–	–	1	1	–	–	2	4 (2.0)
Nasal Swab	–	–	–	–	1	–	–	–	1 (0.5)
Pleural Fluid	–	–	–	1	–	–	1	–	2 (1.0)
Pus	–	3	–	–	–	–	1	1	5 (2.5)
Sputum	–	3	3	3	8	3	2	2	24 (12.0)
Urethral Swab	2	–	–	–	–	–	–	–	2 (1.0)
Urine	6	35	5	23	12	4	4	5	94 (47.0)
Wound Swab	–	4		6	16	–	3	16	45 (22.5)
WARD/DEPT									
A &E	1	2	–	2	2	1	1	1	10 (5.0)
Child Health	1	12	3	11	3	–	1	8	39 (19.5)
Medical ICU	2	14	6	10	21	5	4	16	78 (39.0)
OBS &GYN	2	3	2	4	2	1	1	2	17 (8.5)
OPD	2	12	1	9	5	1	3	6	39 (19.5)
Surgery	–	6	–	2	6	–	–	3	17 (8.5)
Diagnosis									
Abscess	–	1	1	1	1	1	–	2	7 (3.5)
Appendicitis	–	1	–	–	–	–	–	–	1 (0.5)
Bacteremia	–	1	–	–	–	–	–	–	1 (0.5)
Bronchitis	–	–	–	1	1	–	–	–	2 (1.0)
Cellulitis	–	–	–	–	–	–	–	1	1 (0.5)
Cirrhosis	–	1	1	–	–	–	–	–	2 (1.0)
Cystitis	–	–	–	1	–	–	–	–	1 (0.5)
Diabetic foot ulcer	–	1	1	–	4	–	1	3	10 (5.0)
Gastroenteritis	–	4	–	1	–	–	–	–	5 (2.5)
Nephritis	–	6	–	3	–	1	–	1	11 (5.5)
Otitis	–	1	–	–	–	–	–	1	2 (1.0)
Pericarditis	–	1	–	–	2	–	–	–	3 (1.5)
Peritonitis	–	1	–	–	1	–	–	–	2 (1.0)
Pneumoniae	–	–	–	1	–	2	1	–	4 (2.0)
Prostitis	–	–	–	–	–	1	–	–	1 (0.5)
RTI	–	–	–	1	1	–	2	1	5 (2.5)
Sepsis	1	6	1	4	4	2	3	8	29 (14.5)
Surg. site infection	–	1	–	4	4	–	–	1	10 (5.0)
Tuberculosis	–	1	4	2	**7**	1	1	2	18 (9.0)
UTI	7	24	4	17	9	–	2	6	69 (34.5)
Ulcer	–	–	–	–	1	–	–	1	2 (1.0)
Wound infection	–	–	–	1	4	–	–	7	12 (6.0)
Frequency (%)	**8 (4.0)**	**49 (24.5)**	**12 (6.0)**	**38 (19.0)**	**39 (19.5)**	**8 (4.0)**	**10 (5.0)**	**36 (18.0)**	**200 (100)**

*Abbreviation*: *A&E* Accident and Emergency, *OPD* Out-Patient Department, *OBS&GYN* Obstetrics and Gynecology, Other* Other Gram-negative bacteria. The In-Patient Department comprises of A&E, Medical ICU, OBS&GYN and Surgical ward, −: Non-detected

### Demographic characteristics of patients with bacterial infections

The patients’ ages ranged between 2 months to 90 years and mean age was 35.95 ± 27.11 years with the gender distribution of 101 (50.5%) males and 99 (49.5%) females. Samples from outpatients (OPD) made up 19.5% of the total samples. All the other samples (80.5%) from Accident and Emergency, Child Health, Medical ICU, Surgery and Obstetrics and Gynaecology departments were inpatient samples (Table 1). The prevalence of infections were highest among the patients of age-group ≥60 years 49 (24.5%) followed by < 10 years 48 (24.0%), 20–29 years 26 (13.0), 30–39 years and least in 50–59 years 17 (8.5%) (Table 2). UTI was highest among the age-group < 10 years 23 (33.3%) followed by 30–39 years 12 (17.4%) and least 50–59 years 4 (5.8%). Septic infection was highest in patients within the age-group < 10 years 8 (40.0%), followed by ≥60 years 5 (25.0%) least in 10–19 years 3 (15.0%) (Table 2). Among the different types of wound infections, diabetic foot ulcer showed high proportion within age-groups 50–59 and ≥60 years of patients.

**Table 2:** Distribution of infections among gender and patients’ age groups

	Age (Years) Number of Cases (*N*)							
	< 10	10–19	20–29	30–39	40–49	50–59	≥60	Total (%)
Gender								
Female	21	8	20	13	11	9	17	99 (49.5)
Male	27	11	6	8	9	8	32	101 (50.1)
Infections								
Abscess	2	1	2	1	–	1	–	7 (3.5)
Appendicitis	–	1	–	–	–	–	–	1 (0.5)
Bacteremia	1	–	–	–	–	–	–	1 (0.5)
Bronchitis	1	–	–	–	–	–	1	2 (1.0)
Cellulitis	–	–	–	–	–	–	1	1 (0.5)
Cirrhosis	–	1	1	–	–	–	–	21.0)
Cystitis	1	–	–	–	–	–	–	1 (0.5)
Diabetic foot ulcer	–	–	1	–	1	1	7	10 (5.0)
Gastroenteritis	4	3	–	–	–	–	–	7 (3.5)
Nephritis	–	1	1	1	1	1	6	11 (3.5)
Otitis	1	–	1	–	–	–	–	2 (1.0)
Pericarditis	–	–	–	–	2	–	1	3 (1.5)
Peritonitis	2	–	–	–	–	–	–	2 (1.0)
Pneumoniae	–	1	–	–	–	–	3	4 (2.0)
Prostitis	–	–	–	–	–	–	1	1 (1.0)
RTI	2	1	–	1	1	–	–	5 (2.5)
Sepsis	8	3	1	–	1	2	5	20 (10.0)
Surgical wound infection	1	1	2	2	–	2	2	10 (5.0)
Tuberculosis	1	1	3	–	6	3	4	18 (9.0)
UTI	23	4	10	12	7	4	9	69 (34.5)
Ulcer	–	1	–	–	1	–	–	2 (1.0)
Wound infection	1	–	2	2	1	2	2	10 (5.0)
Frequency (%)	**48 (24.0)**	**19 (9.5)**	**26 (13.0)**	**21 (10.5)**	**20 (10.0)**	**17 (8.5)**	**49 (24.4)**	**200 (100)**

*Abbreviation*: *UTI* Urinary tract infection, *RTI* Respiratory tract infection, −: Non-detected

### Susceptibility profile

The antibiotic susceptibility profile showed that the isolates were most resistant to ampicillin (94.4%), trimethoprim/sulfamethoxazole (84.5%), cefuroxime/Axetil (80.0%), cefuroxime (79.0%), cefotaxime (71.3%), cefoxitin (57.5%) and were least resistant to ertapenem (1.5%) (Table 3).

**Table 3:** Antibiotic susceptibility profile of isolates

Antibiotics	Total number of isolates (*N*)	Susceptible *N* (%)	Intermediate *N* (%)	Resistant *N* (%)
Ampicillin	162	7 (4.3)	2 (1.2)	153 (94.4)
Amox/clav	200	51 (25.5)	47 (23.5)	102 (51.5)
Piperacillin-tazobactam	198	109 (54.5)	62 (31.0)	27 (13.5)
Cefuroxime	200	35 (17.5)	7 (3.5)	158 (79.0)
Cefoxitin	200	74 (37.0)	11 (5.5)	115 (57.5)
Cefotaxime	199	44 (22.1)	12 (6.0)	143 (71.3)
Ceftazidime	200	104 (52.0)	15 (7.5)	81 (40.5)
Cefepime	200	119 (59.5)	64 (32.0)	17 (8.5)
Ertapenam	132	130 (98.5)	1 (0.8)	1 (0.8)
Imipenem	199	191 (95.5)	0 (0.0)	8 (4.0)
Meropenem	198	192 (97.0)	1 (0.5)	5 (2.5)
Amikacin	199	178 (89.4)	14 (7.0)	7 (3.5)
Gentamicin	198	105 (53.0)	5 (2.5)	88 (44.4)
Ciprofloxacin	200	116 (58.0)	2 (1.0)	82 (41.0)
Tetracycline	199	124 (62.3)	14 (7.0)	61 (30.7)
Nitrofuratoin	200	84 (42.0)	17 (8.5)	99 (49.5)
Collistin	198	164 (82.8)	2 (1.0)	32 (16.2)
Trim/Sulfamethoxazole	200	31 (15.5)	0 (0.0)	169 (84.5)

Not all the antibiotics were tested for all 200 isolates and not all *N*-values added up to 200

*Abbreviation*: *amox/clav* amoxicillin-clavulanate, *trim/sulfamethoxazole* trimethoprim-sulfamethoxazole

#### Multi-drug resistance

Multidrug resistance was observed in 89.5% of the bacterial isolates, ranging from 53.8% in *Enterobacter* spp. to 100.0% in *Acinetobacter* spp. and *P. aeruginosa* (Table 4).

**Table 4:** MDR among Isolates

Bacterial Isolates	Number of Isolates (*N*)	MDR *N* (%)
*Acinetobacter spp*	8	8 (100.0)
*E. coli*	49	44 (89.9)
*Enterobacter spp*	12	7 (53.8)
*K. pneumoniae*	38	36 (94.7)
*P. aeruginosa*	39	39 (100.0)
*Serratia spp*	8	7 (87.5)
*Sphingomonas paucimobilis*	10	9 (90.0)
Other Gram negative	36	29 (80.6)
Frequency	**200**	**179 (89.5)**

## Discussion

Epidemiological surveillance of bacterial infection and resistance to antibiotics are essential for awareness creation, implementation of control measures and effective management of infections. This is important in developing countries particularly in sub-Saharan Africa where studies have indicated that many hospitals have rudimentary and poor enforcement of infection control measures and marginal awareness on the extent of infections caused by multi-drug resistant bacteria which have resulted in increased morbidity and mortality [10, 11].

Our study observed higher prevalence of infections among in-patients (80.5%) compared to the out-patients (19.5%), with ICU accounted for highest incidence. The frequency of bacterial pathogens isolated from male (50.5%) and female (49.5%) patients, showed no appreciable difference. The high infections of in-patients is consistent with the finding from nationwide surveillance on antimicrobial resistant pathogens from patients’ blood cultures, which recorded prevalence of > 70% of bacterial infections among in-patients in Ghanaian hospitals [12]. Several predisposing factors are associated with the higher infection rates among hospitalized patients such as the use of invasive procedures like catheterization, central lines and mechanical ventilation [13]. A number of risk factors account for the high ICU infections including non-compliance of care professionals (physicians and nurses) to hand-hygiene practices which has been identified in a study conducted in a neonatal ICU in a Ghanaian tertiary care hospital as a major factor contributing to healthcare infections in ICU [14]. The ICU houses critically ill patients and are often exposed to extensive use of antibiotics causing selection pressure for the emergence of resistance. These factors coupled with interventional instrumentations such as mechanical ventilation and invasive procedures, commonly used in ICU [15], exposes the patients to high risk of infections. KATH is the only tertiary and referral facility receiving patients into the ICU from many healthcare facilities within the region and other parts of the country, often resulting in congestion or overcrowding, thus increasing chances of transmitting infections among patients.

This study indicated high infections among advancing and infant age-groups with UTI and sepsis recorded as most frequent. The geriatric and pediatric patients are usually more disposed to infections due to their immune status. The advancing age are commonly associated with risk factors including reduced immunity, co-morbid diseases such as diabetes mellitus, chronic heart diseases, neurogenic bladder [13, 16] whilst in infants, lack of fully developed immunity, malnutrition as well as inadequate hygiene [17] put them at greater risk of infections. Urinary tract infection (34.5%) was most prevalent within the period of our study, and is comparable to 31.5% reported from a study on prevalence and antibiotic susceptibility pattern of uropathogens conducted in secondary hospital in Ghana [18]. Our study found, *E. coli* and *K. pneumoniae* as most predominant pathogens implicated in UTI. Several studies conducted in the region and other parts of the country have reported UTI as most common infections frequently caused by *E.coli* and *K. pneumoniae* with high resistance to broad spectrum antibiotics, that remains a major clinical problem in health care system in Ghana [19, 20]. Among the isolates, *E. coli*, *K. pneumoniae Proteus mirabilis*, *P. aeruginosa*, *Enterobacter*, *Acinetobacter* and *Serratia* spp. in the present study have been reported as clinically important urine pathogens [18, 21], associated with about 90% of both community and hospital acquired UTIs [22, 23]. Urinary tract infection prevalence was high among infants and the middle aged with incidence higher in females (10.8%) than the males (6.6%) in the middle age group. In infants predisposing factors such as lack of personal hygiene, incomplete emptying of the bladder with residual urine and severe acute malnutrition have been reported [24], whilst the middle aged, in particular females, high parity coupled with increased frequency of sexual activities have been identified, contributing to the high incidence [25]. The prevalence of sepsis among infants, 40.0% (Table 2), was higher compared to the 25.9% previously reported in the country [26]. The higher prevalence is attributed to higher patients’ intake in the study site and also situated in most populous region, thus receiving higher numbers of patients (infants) with complications compared to other regions of Ghana. This study is however limited in its representativeness of the entire hospital because we did not systematically collect data on how much of each specimen type was sent to the lab and which types of isolates were obtained from each specimen type.

Among the isolates, *E. coli*, *P. aeruginosa* and *K. pneumoniae* were most prevalent pathogens implicated in 63.0% (Table 1) of the infections with high resistance to antibiotics commonly used in Ghana. The finding that *E coli*, *P. aeruginosa* and *K. pneumoniae* were most prevalent Gram-negative pathogens is consistent with previous studies conducted in Ashanti region of Ghana [20] and other parts of the country [26, 27]. A nationwide surveillance on antimicrobial resistant pathogens study, conducted by Opintan et al. also indicated *E.coli* and *P. aeruginosa*, *Enterobacter*, *Citrobacter spp.* and *K. pneumoniae* as most common gram-negative bacterial pathogens in Ghana [28]. The current prioritized lists of bacterial pathogens by World Health Organization (WHO), categorized *E. coli*, *P. aeruginosa*, *K. pneumoniae*, *Acinetobacter*, *Enterobacter*, *Serratia*, *Proteus* and *Providencia* spp. identified in our study, as critical or most life-threatening Gram-negative pathogens under surveillance due to their high antibiotic resistance especially to carbapenems and third generation cephalosporins, associated with attributable mortality [29]. In particular, *K. pneumoniae*, *P. aeruginosa*, *Acinetobacter* and *Enterobacter* spp. have further been described by the Infectious Diseases Society of America as Gram-negative ESKAPE (*Enterococcus faecium*, *Staphylococcus aureus*, *Klebsiella pneumoniae*, *Acinetobacter baumannii*, *Pseudomonas aeruginosa* and *Enterobacter* spp) pathogens, frequently associated with multidrug resistance [30, 31]. These ESKAPE Gram-negative pathogens were implicated in 48.5% (Table 1) of the infections which is higher than 35.6% previously reported [27]. The pathogens were also implicated in 50% (Table 1) of the ICU infections, and therefore posing a major threat to public healthcare in Ghana.

The susceptibility profile of the isolates displayed high level multidrug resistance of 89.5% with high resistance to ampicillin (94.4%), trimethoprim sulfamethoxazole (84.5%), cefuroxime (79.0%) cefotaxime (71.3%), cefoxitin (57.5%) and amoxacillin-clavulanate (51.5%) observed, was consistent with other studies conducted in Ghana [27, 32]. The degree of resistance among the isolates to ampicillin, trimethoprim/sulfamethoxazole and amoxacillin-clavulanate showed in the study, also agreed with other studies conducted in the sub-Sahara African countries such as Tanzania [7], Nigeria [33], Ethiopia [34], Zimbabwe [35] and Rwanda [6]. The high resistance trend in the sub region is indicative of high antibiotic selection pressure largely due to relatively cheap and easy availability of these agents, mostly used as first line or common choice of treatment in many healthcare settings in the sub region [36–38].

The decreased susceptibility of isolates to the beta-lactam/beta-lactamases inhibitor combination antibiotic therapy and fluoroquinolones (ciprofloxacin) is comparable to other studies in Ghana [20, 27], which poses a challenge to treatment of common infections as these agents are readily available therapeutic options [39]. The high resistance to second and third-generation cephalosporins (cefuroxime [79.0%], cefoxitin [57.5%] and cefotaxime [71.3%]), may suggest high expression or production of extended spectrum beta-lactamases among Gram-negative bacteria as previously reported in Ghana [40]. The carbapenems (imipenem, ertapenem and meropenem), amikacin and colistin were sensitive, as these antibiotics are used as last resort in treatment of serious infections. In addition the carbapenems have been introduced into Ghanaian market for relatively short span of time and is comparatively more expensive than the mainstay antibiotics and therefore, not commonly used.

This has possibly led to relatively low natural selection and hence low development of antibiotic resistance among the isolates.

## Conclusion

The study demonstrated high multidrug resistant Gram-negative bacteria implicated in the infections, with UTI as most frequently diagnosed among patients. Infections were common among the elderly and infants, and predominantly caused by *E. coli*, *K. pneumoniae* and *P*. *aeruginosa* during the period of our study. These results should inform the empirical treatment of infections in Komfo Anokye Teaching Hospital of Ghana as appropriate.

## References

[1] Singh N, Manchanda V (2017). Control of multidrug-resistant gram-negative bacteria in low-and middle-income countries—high impact interventions without much resources. Clin Microbiol Infect.

[2] De Angelis G, D’Inzeo T, Fiori B, Spanu T, Sganga G (2014). Burden of antibiotic resistant gram negative bacterial infections: evidence and limits. J Med Microbiol Diagn.

[3] Rossolini GM, Mantengoli E, Docquier J, Musmanno RA, Coratza G (2007). Epidemiology of infections caused by multiresistant gram-negatives: ESBLs, MBLs, panresistant strains. New Microbiol.

[4] Oduro-Mensah D, Obeng-Nkrumah N, Bonney EY, Oduro-Mensah E, Twum-Danso K, Osei YD, Sackey ST (2016). Genetic characterization of TEM-type ESBL-associated antibacterial resistance in Enterobacteriaceae in a tertiary hospital in Ghana. Ann Clin Microbiol Antimicrob.

[5] Le Doare K, Bielicki J, Heath PT, Sharland M (2015). Systematic review of antibiotic resistance rates among gram-negative bacteria in children with sepsis in resource-limited countries. J Pediatric Infect Dis Soc.

[6] Carroll M, Rangaiahagari A, Musabeyezu E, Singer D, Ogbuagu O (2016). Five-year antimicrobial susceptibility trends among bacterial isolates from a tertiary health-care facility in Kigali, Rwanda. Am J Trop Med Hyg.

[7] Kumburu HH, Sonda T, Mmbaga BT, Alifrangis M, Lund O, Kibiki G, Aarestrup FM (2017). Patterns of infections, aetiological agents, and antimicrobial resistance at a tertiary care hospital in northern Tanzania. Tropical Med Int Health.

[8] Allegranzi B, Nejad SB, Combescure C, Graafmans W, Attar H, Donaldson L, Pittet D (2011). Burden of endemic health-care-associated infection in developing countries: systematic review and meta-analysis. Lancet.

[9] Owusu G, Oteng-Ababio M (2015). Moving unruly contemporary urbanism toward sustainable urban development in Ghana by 2030. Am Behav Sci.

[10] Huttner A, Harbarth S, Carlet J, Cosgrove S, Goossens H, Holmes A, Jarlier V, Voss A, Pittet D (2013). Antimicrobial resistance: a global view from the 2013 World Healthcare-Associated Infections Forum. Antimicrob Resist Infect Control.

[11] Samuel S, Kayode O, Musa O, Nwigwe G, Aboderin A, Salami T, Taiwo S (2010). Nosocomial infections and the challenges of control in developing countries. Afr J Clin Exp Microbiol.

[12] Opintan JA, Newman MJ (2017). Prevalence of antimicrobial resistant pathogens from blood cultures: results from a laboratory based nationwide surveillance in Ghana. Antimicrob Resist Infect Control.

[13] Chang YJ, Yeh ML, Li YC, Hsu CY, Lin CC, Hsu M-S, Chiu WT (2011). Predicting hospital-acquired infections by scoring system with simple parameters. PLoS One.

[14] Asare A, Enweronu-Laryea CC, Newman MJ (2009). Hand hygiene practices in a neonatal intensive care unit in Ghana. J Infect Dev Ctries.

[15] Ulu-Kilic A, Ahmed S, Alp E, Doğanay M (2013). Challenge of intensive care unit-acquired infections and Acinetobacter baumannii in developing countries. Crit Care.

[16] Simonetti AF, Viasus D, Garcia-Vidal C, Carratalà J (2014). Management of community-acquired pneumonia in older adults. Ther Adv Infect Dis.

[17] Hill R, Paulus S, Dey P, Hurley MA, Carter B (2016). Is undernutrition prognostic of infection complications in children undergoing surgery? A systematic review. J Hosp Infect.

[18] Gyansa-Lutterodt M, Afriyie D, Asare G, Amponsah S, Abutiate H, Darko D (2014). Antimicrobial use and susceptibility pattern of uropathogens associated with urinary tract infections at the Ghana Police Hospital. Glob J Pharmacol.

[19] Obirikorang C, Quaye L, Bio F, Amidu N, Acheampong I, Addo K (2012). Asymptomatic bacteriuria among pregnant women attending antenatal clinic at the University Hospital, Kumasi, Ghana. J Med Biomed Sci.

[20] Gyasi-Sarpong CK, Nkrumah B, Yenli EMT-A, Appiah AA, Aboah K, Azorliade R, Kolekang AS, Ali I (2014). Resistance pattern of uropathogenic bacteria in males with lower urinary tract obstruction in Kumasi, Ghana. Afr J Microbiol Res.

[21] Wireko S, Abubakari A, Opoku B (2017). In vitro activities of antimicrobial agents against uropathogenic isolates at Brong Ahafo regional hospital, Ghana. Int J Curr Microbiol App Sci.

[22] Linhares I, Raposo T, Rodrigues A, Almeida A (2013). Frequency and antimicrobial resistance patterns of bacteria implicated in community urinary tract infections: a ten-year surveillance study (2000–2009). BMC Infect Dis.

[23] Farajnia S, Alikhani MY, Ghotaslou R, Naghili B, Nakhlband A (2009). Causative agents and antimicrobial susceptibilities of urinary tract infections in the northwest of Iran. Int J Infect Dis.

[24] Uwaezuoke SN (2016). The prevalence of urinary tract infection in children with severe acute malnutrition: a narrative review. Pediatric Health Med Ther.

[25] Komala M, Kumar KS (2013). Urinary tract infection: causes, symptoms, diagnosis and it’s management. Indian J Res Pharm Biotechnol.

[26] Acquah SE, Quaye L, Sagoe K, Ziem JB, Bromberger PI, Amponsem AA (2013). Susceptibility of bacterial etiological agents to commonly-used antimicrobial agents in children with sepsis at the Tamale Teaching Hospital. BMC Infect Dis.

[27] Newman MJ, Frimpong E, Donkor ES, Opintan JA, Asamoah-Adu A (2011). Resistance to antimicrobial drugs in Ghana. Infect Drug Resist.

[28] Opintan JA, Newman MJ, Arhin RE, Donkor ES, Gyansa-Lutterodt M, Mills-Pappoe W (2015). Laboratory-based nationwide surveillance of antimicrobial resistance in Ghana. Infect Drug Resist.

[29] WHO (2017). Global priority list of antibiotic-resistant bacteria to guide research, discovery, and development of new antibiotics.

[30] Navidinia M (2016). The clinical importance of emerging ESKAPE pathogens in nosocomial infections. J Paramed Sci.

[31] Cerceo E, Deitelzweig SB, Sherman BM, Amin AN (2016). Multidrug-resistant gram-negative bacterial infections in the hospital setting: overview, implications for clinical practice, and emerging treatment options. Microb Drug Resist.

[32] Obeng-Nkrumah N, Twum-Danso K, Krogfelt KA, Newman MJ (2013). High levels of extended-spectrum beta-lactamases in a major teaching hospital in Ghana: the need for regular monitoring and evaluation of antibiotic resistance. Am J Trop Med Hyg.

[33] Ogbolu D, Daini O, Ogunledun A, Alli A, Webber M (2011). High levels of multidrug resistance in clinical isolates of gram-negative pathogens from Nigeria. Int J Antimicrob Agents.

[34] Muluye D, Wondimeneh Y, Ferede G, Nega T, Adane K, Biadgo B, Tesfa H, Moges F (2014). Bacterial isolates and their antibiotic susceptibility patterns among patients with pus and/or wound discharge at Gondar university hospital. BMC Res Notes.

[35] Mbanga J, Dube S, Munyanduki H (2010). Prevalence and drug resistance in bacteria of the urinary tract infections in Bulawayo province, Zimbabwe. East Afr J Public Health.

[36] Mshana SE, Matee M, Rweyemamu M (2013). Antimicrobial resistance in human and animal pathogens in Zambia, Democratic Republic of Congo, Mozambique and Tanzania: an urgent need of a sustainable surveillance system. Ann Clin Microbiol Antimicrob.

[37] Ocan M, Bwanga F, Bbosa GS, Bagenda D, Waako P, Ogwal-Okeng J, Obua C (2014). Patterns and predictors of self-medication in northern Uganda. PLoS One.

[38] Donkor ES, Tetteh-Quarcoo PB, Nartey P, Agyeman IO (2012). Self-medication practices with antibiotics among tertiary level students in Accra, Ghana: a cross-sectional study. Int J Env Res Public Health.

[39] Hackman HK, Osei-Adjei G, Ameko E, Kutsanedzie F, Gordon A, Laryea E, Quaye S, Anison L, Brown CA, Twum-Danso K (2013). Phenotypic determination and antimicrobial resistance profile of extended spectrum beta-lactamases in Escherichia coli and Klebsiella pneumoniae in Accra, Ghana. J Nat Sci.

[40] Feglo P, Adu-Sarkodie Y (2016). Antimicrobial resistance patterns of extended spectrum Β-lactamase producing Klebsiellae and E. coli isolates from a tertiary hospital in Ghana. Eur Sci J.

